# Serum Interleukin-19 Levels in Acne Vulgaris Patients of Varying Clinical Severity in Erbil City

**DOI:** 10.7759/cureus.48939

**Published:** 2023-11-17

**Authors:** Hazha M Mohammed, Dindar S Qurtas, Alan D Meran

**Affiliations:** 1 Dermatology, Erbil Dermatology Teaching Center, Erbil, IRQ; 2 College of Medicine, Hawler Medical University, Erbil, IRQ

**Keywords:** erbil, inflammation, global acne score index, il-19, acne vulgaris

## Abstract

Background

Acne vulgaris (AV) is a common multifactorial disorder affecting the pilosebaceous units. Research has shown that inflammation plays a crucial role in the pathogenesis of AV, including both inflammatory and non-inflammatory acne. Several studies have linked proinflammatory cytokines to AV; however, only a few have explored the correlation between interleukin-19 (IL-19) and AV. Our aim is to estimate the level of IL-19 in patients with AV compared to matched controls and to investigate the role of IL-19 in the pathogenesis of acne.

Materials and methods

This prospective cross-sectional case and control study includes 80 patients and 40 matched controls. Patients were divided into mild (20), moderate (40), and severe (20) groups based on their global acne score severity index. Detailed history and complete general and dermatological examinations were taken from each patient. Furthermore, 5 ml of blood was taken from all participants to assess the level of IL-19.

Results

IL-19 levels were significantly higher in patients with AV compared to matched controls. Furthermore, IL-19 concentrations were found to be proportional to the severity of acne, with the highest levels detected in patients with severe AV (p-value <0.005).

Conclusion

IL-19 levels in AV were significantly higher than in matched control. The difference was proportional to its severity. This might indicate IL-19 as an inflammatory marker and could potentially be related to AV.

## Introduction

A prevalent, long-lasting inflammation of the sebaceous glands is known as acne vulgaris (AV). It is a common disorder mainly affecting adolescents and young adults. Approximately 27%-93% of teens and late adolescents are affected by it [1]. AV preferentially affects the face, chest, and upper back with the appearance of comedones. Inflamed lesions are characterized by red papules, pustules, and often subcutaneous cysts. Scarring, either atrophic, hypertrophic, or keloid formation, can occur even after the disappearance of inflammation [2]. The pathogenesis of AV is multifactorial in its origin, of which the key factor is increased sebum production, inflammatory processes, follicular hyperproliferation, and the proliferation of Cutibacterium acnes (C. acnes) [3]. Inflammatory reactions occur in all phases of the development of acne lesions, which is confirmed by histopathological and immunologic studies; for that reason, inflammation has a crucial role in forming both inflammatory and noninflammatory lesions in AV [4]. The proliferation of C. acnes in AV patients stimulates keratinocytes upon the bacteria's binding to Toll-like receptors (TLRs). This results in the production of proinflammatory cytokines such as IL-1, which subsequently induces the expression of Interleukin-19 (IL-19) in keratinocytes both in vitro and in vivo [5]. So far, many other cytokines have been found to be related to the pathogenesis of AV, including IL-6, IL-8, IL-10, and IL-12 [6]. The IL-19 human genetic locus is located on human chromosome 1q32, and this locus has a strong link to IL-10 as part of a gene cluster [7]. When exposed to proinflammatory stimuli, monocytes and epithelial cells produce IL-19 [8, 9]. In turn, IL-19 amplifies the proinflammatory nature by creating a positive feedback loop, encouraging these cells to further amplify their response. As soon as they are stimulated in the inflammatory process, they will continuously produce the cytokine [10, 11]. Recently, numerous reports have focused on adiponectin levels and their association with AV. It has been found that patients with AV typically have lower levels of adiponectin, which can boost the production of proinflammatory cytokines and escalate the pathogenesis [12, 13]. There are few international studies investigating IL-19 concentration in AV patients. Mochtar M et al., in their study, stated that there were changes in IL-19 concentration in AV patients at different stages of clinical severity. They suggested that IL-19 concentration levels may play a role in the pathophysiology of AV and be associated with its degree of clinical severity [14]. However, similar national-level studies on the link between IL-19 cytokines and AV or its severity are lacking. Therefore, our study primarily aims to examine the variation in IL-19 serum levels between AV patients and controls, as well as to investigate the differences in IL-19 serum levels among AV patients at different stages of clinical severity. Additionally, the study seeks to explore the impact of sociodemographic criteria and their relationship with the modulation of IL-19 concentration and the outcomes in AV patients.

## Materials and methods

This prospective cross-sectional case-control study included 120 subjects aged between 12 and 39 years who were recruited from the Erbil Dermatology Teaching Center in Erbil City, Iraq, between April 2022 and August 2022. The control group comprised individuals visiting the same clinic for other conditions. Study participants were allocated into three groups: mild, moderate, and severe AV patient groups, based on their acne severity, and an apparently healthy control group of matched age and sex with no prior history of acne or active acne. Eligibility criteria for patients included being between 12 and 35 years old, having a confirmed clinical diagnosis of AV, and willingness to participate in the research, including completing a questionnaire and a declaration of willingness to participate in the study. Exclusion criteria encompassed AV patients who had undergone systemic or topical treatment in the last two months and two weeks, respectively, as well as pregnant or postpartum women. Additionally, patients with other diseases potentially affecting IL-19 serum expression, such as psoriasis, atopic dermatitis, and asthma, were also excluded [15-17].

Ethical considerations

Our study received approval from the local ethics committee of the Kurdistan Higher Council for Medical Specialties. The patients agreed to participate in the study and filled out informed consent forms, after which the participants were assigned as follows.

History taking

A detailed history was obtained from each participant, including information about their name, age, sex, onset of the disease, skin type, family history of acne, history of medical diseases, and drug intake.

Clinical examination

A detailed general and dermatological examination was conducted for all participants to identify the type of lesions, which could be inflammatory (papules, pustules, nodules) or non-inflammatory (comedones). The distribution of lesions (face, back, and/or shoulders) and the presence or absence of scarring or hyperpigmentation were also noted. Patients were additionally screened for any exclusion criteria, such as psoriasis. For the assessment of acne severity, we used the Global Acne Grading System (GAGS). The scoring calculation is based on the location and type of the lesion [18]. Based on the results of this calculation, patients were categorized into mild (1-18), moderate (19-30), severe (31-39), and very severe (>39).

Measurement of serum IL-19 levels

Sample Collection and Storage

Under full aseptic conditions, 5 mL of venous blood samples were collected from all cases and controls and then transferred into plain tubes. These were left for 30 minutes at 37°C to allow clotting, followed by centrifugation for 10 minutes at 3000 RPM. The obtained serum was preserved for the assessment of IL-19 levels using enzyme-linked immunosorbent assay (ELISA). The serum IL-19 concentration was measured using quantitative human IL-19 ELISA kits (Elabscience Biotechnology Inc., Wuhan, China).

Assay Procedure

All components, standard solutions, and samples were prepared as advised, based on the manufacturer's recommendations [19]. 100μL of the standard or sample was added to each well and incubated for 90 minutes at 37°C. Afterward, the liquid was discarded, and 100μL of biotinylated detection antibody working solution was added to each well, followed by another incubation for 60 minutes at 37°C. The liquid was then discarded, and the wells were washed three times. Next, 100μL of a horseradish peroxidase (HRP) conjugate working solution was added to each well, and the wells were incubated in the dark for 30 minutes at 37°C. This was followed by aspirating the liquid and washing the plate five times. Subsequently, 90μL of substrate reagent was added to each well and incubated for 15 minutes at 37°C. The reaction was stopped by adding 50μL of stop solution. The optical density (OD) of each well was measured using a plate reader set at 450 nm directly after adding the stop solution.

Statistical analysis

All findings were statistically assessed using SPSS version 23 (IBM Corp., Armonk, NY, USA). Descriptive statistics, including mean and standard deviation for numeric data and percentages for qualitative data, were calculated. For analytical statistics, the Chi-square test (χ2) and Mann-Whitney test (U) were performed to examine the relationship between qualitative and quantitative variables, respectively. The Kruskal-Wallis test was utilized to compare the mean IL-19 levels among different groups of acne patients with varying clinical severity. A p-value of less than 0.005 was considered significant.

## Results

Clinical characteristics of the study group

We included 120 participants, comprising 80 cases of AV and 40 non-acne controls with matched age and gender. The AV patients were divided into three groups based on Global Acne Grading System (GAGS) scores: mild (n=20, 25%), moderate (n=40, 50%), and severe (n=20, 25%) (Table 1). The mean age of the participants was 19.9 years, ranging from 12 to 35 years. Participants were categorized into three age groups: 12-18 years, 19-30 years, and over 30 years. A negative but not significant relationship was observed between age and acne severity (P=0.059), indicating that increased age was associated with milder acne (Table 1). Females (n=68, 57%) slightly outnumbered males, with no significant impact on acne outcomes. However, a significant gender difference was noted in severe cases, with a higher prevalence among males (11/52, 21% vs. 9/68, 13.2%) (Table 1). Regarding clinical data, most cases had a progressive onset (n=64, 80%), and a positive family history of acne was noted in 65% of the patients (n=45) compared to 28% in controls, showing a significant difference. Additionally, most cases with a positive family history had moderate acne severity. Severe acne was also significantly associated with skin scarring (Table 1).

**Table 1:** Sociodemographic characteristics of patients according to the severity of acne.

Variables		Control (%)	Mild (%)	Moderate (%)	Severe (%)	Total	P-value
	Patient Number	N=40 (34%)	N=20 (17%)	N=40 (34%)	N=20 (17%)		
Gender	Male	17 (32.7)	8 (15.4%)	16 (30.8%)	11 (21.2%)	52 (100%)	0.474
	Female	23 (33.8%)	12 (17.6%)	24 (35.3%)	9 (13.2%)	68 (100%)	
Age	12-18	15 (37.5%)	8 (40%)	19 (47.5%)	15 (75%)	57 (100%)	0.059
	19-30	18 (45%)	11 (55%)	21 (52.5%)	5 (25%)	55 (100%)	
	>31	7 (17.5%)	1 (5%)	0 (%)	0 (0%)	8 (100%)	
Family history	Positive	9 (16.7%)	12 (22.2%)	25 (46.3%)	8 (14.8)	54 (100%)	** 0.016
	Negative	23 (43.4%)	5 (9.4)	13 (24.5%)	12 (22.6%)	53 (100%)	
Acne scar	Positive	1 (3.1%)	6 (18.8%)	12 (37.5%)	13 (40.6%)	32 (100%)	*** 0.004
	Negative	36 (43.9%)	14 (17.1%)	25 (30.5%)	7 (8.5%)	82 (100%)	
Treatment received	No	40 (50.5%)	12 (15.2%)	18 (22.8%)	9 (11.4)	79 (100%)	*** <0.001
	Topical	0 (0%)	4 (14.8%)	16 (59.3%)	7 (25.9%)	27 (100%)	
	Systemic	0 (0%)	2 (100%)	0 (0%)	0 (0%)	2 (100%)	
	Both	0 (0%)	2 (16.7%)	6 (50%)	4 (33.3%)	12 (100%)	
Skin color	II	9 (50%)	1 (5.6)	4 (22.2%)	4 (22.2%)	18 (100%)	0.208
	III	28 (31.8%)	19 (21.6%)	25 (28.4%)	16 (18.2%)	88 (100%)	
	IV	3 (23.1%)	0 (0%)	10 (76.9%)	0 (0%)	13 (100%)	

Measurement of serum IL-19 levels

**Figure 1:**
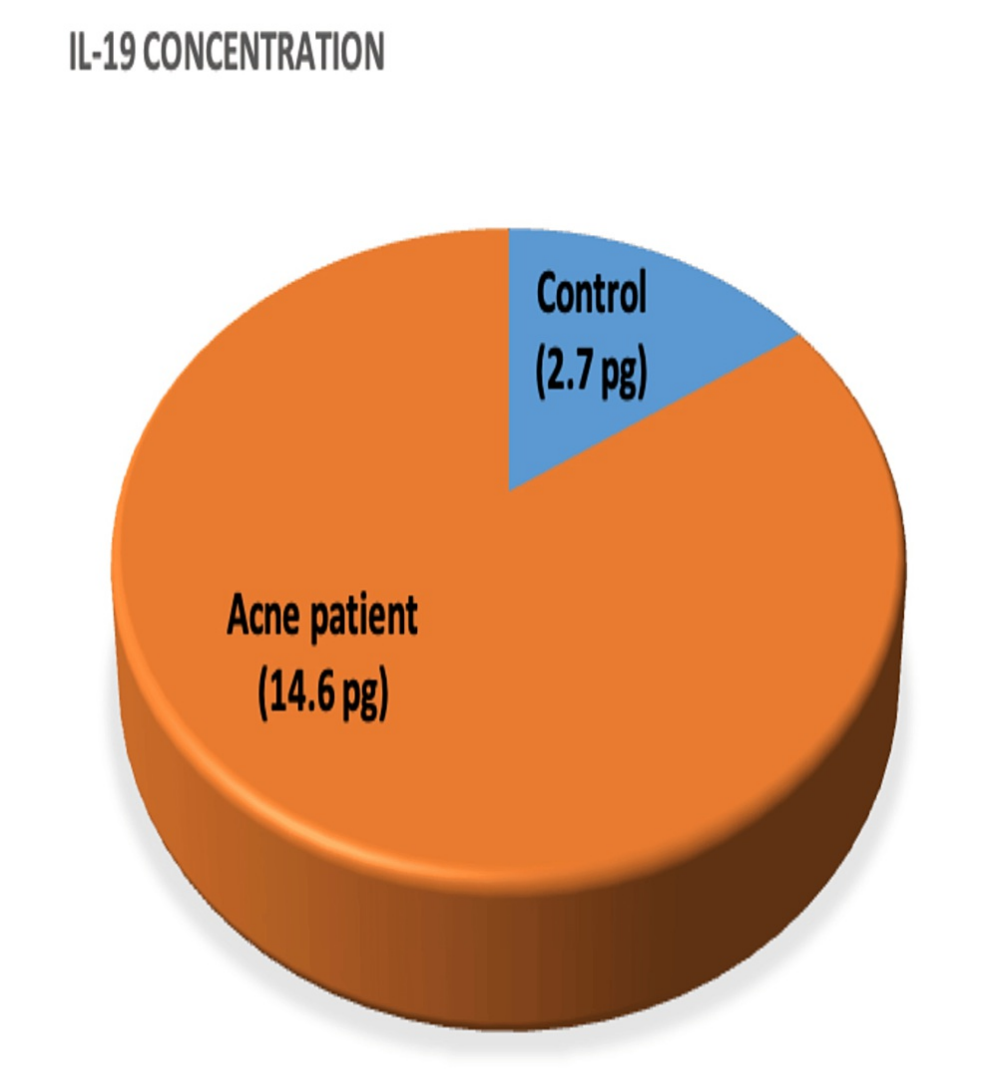
Comparison of IL-19 concentration between patients and control group. IL-19: Interleukin-19.

The serum concentration of IL-19 was measured in all participants, revealing a significant difference in IL-19 levels. A notable disparity was observed between cases and controls (P<0.001), with the mean serum level of IL-19 being higher in cases (14.58) compared to controls (2.68), as demonstrated in Figure 1 and Table 2.

**Table 2:** IL-19 concentration according to the severity of acne and other variables. IL-19: Interleukin-19.

Variable		IL-19 concentration in relation to different variables	
	IL-19 concentration	IL-19 pgm/mL	P-value
Age	12-18 (n=57)	10.203	0.149
	19-30 (n=55)	11.97	
	31 and more (n=8)	5.1263	
Gender	Male (n=52)	10.1533	0.468
	Female (n=68)	11.0726	
Acne Onset	Sudden (n=16)	10.004	0.191
	Progressive (n=64)	15.838	
Acne Severity	Control (n=40)	2.68	<0.003
	Mild (n=20)	8.27	
	Moderate (n=40)	14.939	
	Sever (n=20)	20.536	
Acne scar	Negative (n=53)	11.219	<0.013
	Positive (n=32)	16.89	
Treatment received	No (n=79)	6.989	0.0003
	Topical (n=27)	14.445	
	Systemic (n=2)	20.395	
	Both n=12	24.825	

**Figure 2:**
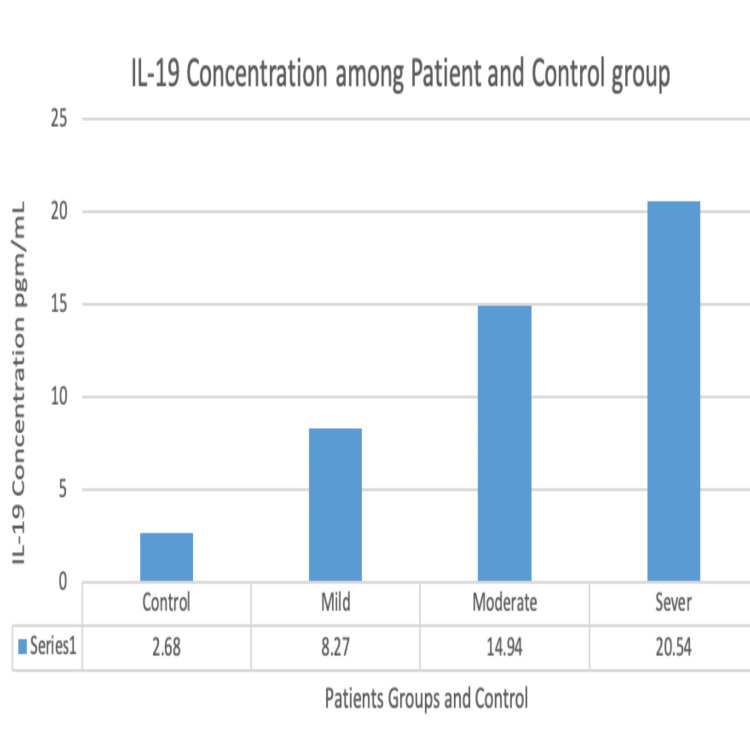
IL-19 concentration in control group and acne patients of varying severity. IL-19: Interleukin-19.

AV patients were categorized using the GAGS based on their AV severity. We further analyzed the IL-19 concentration level among acne groups of different clinical severity levels, based on which we could detect a proportional significant difference between serum concentration of IL-19 and acne severity. The serum concentration of IL-19 was estimated to be 8.27 pg/mL for mild acne cases, 14.94 pg/mL for moderate cases, and 20.53 pg/mL for severe cases (P<0.003), as shown in Figure 2 and Table 2.

We noted that the serum concentration of IL-19 gradually rose with age, reaching its peak at 30 years of age, and then decreased after that; however, these changes were not statistically significant (Table 2).

Most participants experienced a progressive onset of their disease. In our analysis of IL-19 serum concentration, we observed higher levels of IL-19, although the difference was not statistically significant. This finding is still noteworthy. Further analysis revealed a significantly higher IL-19 concentration in patients with acne scars. Additionally, individuals who had received systemic acne treatment within the last two months exhibited elevated IL-19 levels. These levels were highest among those undergoing combined treatment (Table 2).

## Discussion

AV is a disease that affects the pilosebaceous unit and has a complex, multifactorial origin. Recently, inflammation has been regarded as a major player in its pathogenesis, with Propionibacterium acting as an important triggering factor in this regard and having a direct effect on AV severity [20]. The skin's lipid-rich layer acts as a conducive environment for Propionibacterium proliferation, which induces inflammation through the activation of the inflammatory cascade. Recent findings show that Propionibacterium activates TLR-2, followed by the release of certain proinflammatory cytokines such as IL-1β [21]. Other studies have noted the direct effect of IL-1β and IL-19, where the release of IL-1β induces epithelial cells and keratinocytes to express IL-19 both in vitro and in vivo through an unknown mechanism. This is followed by an inflammatory process and might be a triggering factor in increasing the incidence and severity of AV [22, 23]. In our study, we aimed to estimate the IL-19 serum level among patients diagnosed with AV of various degrees of severity, as well as among healthy controls of matched age and sex. Our results showed that IL-19 concentration was significantly higher among AV patients compared to age- and sex-matched controls (Figure 1). Additionally, IL-19 levels significantly differed between mild, moderate, and severe AV patients, with changes being proportional to the degree of clinical severity, as shown in Figure 2 and Table 1. Several other studies assessed the IL-19 concentration level, and the role of IL-19 in the pathogenesis of other skin inflammatory diseases was established by several previous studies for psoriasis and atopic dermatitis [24]. Several other studies have assessed IL-19 concentration levels, and the role of IL-19 in the pathogenesis of other inflammatory skin diseases, such as psoriasis and atopic dermatitis, was established in previous studies [24]. Mochtar M et al., who were the first to investigate IL-19 levels in patients with AV of varying severity, found results similar to ours. They demonstrated a statistically significant difference in IL-19 concentration between mild and severe cases, as well as between moderate and severe cases. However, in contrast to our findings, they did not observe a significant difference between mild and moderate cases [23]. Our results showed significant differences not only between mild and severe and moderate and severe cases but also between mild and moderate cases, a finding also confirmed by another study [25]. This suggests that higher concentrations of IL-19 are associated with more severe inflammation. This is in line with findings by Yahil RJ, who demonstrated that the production of the proinflammatory cytokine IL-19 mainly depends on cells involved in the inflammatory microenvironment, implying that severe inflammation correlates with higher levels of IL-19 [26]. Similarly, our findings are consistent with those of Li HH et al., who estimated IL-19 levels in patients with another inflammatory skin disorder, psoriasis, highlighting that disease severity is reflected in increased levels of proinflammatory cytokines [27]. In our study, the median IL-19 serum concentration among cases of different severity and controls was lower than that found by Mochtar M et al. However, variations in IL-19 concentrations have been reported in similar studies [15, 25], which could be attributed to racial differences or the use of different experimental kits by different investigators.

Additionally, Konrad RJ et al. assessed the level of IL-19 in psoriasis and atopic dermatitis, correlating the cytokine level with disease severity. Using severity indices for both conditions, they found a significant positive correlation between the serum level of IL-19 and disease severity in both psoriasis and atopic dermatitis [22]. They also reported a reduction in IL-19 concentration levels to normal after skin improvement in both conditions following biological therapy (Ixekizumab for psoriasis and Baricitinib for atopic dermatitis) [24]. IL-19 has been shown to be expressed in inflammatory conditions in various diseases [14, 16, 24]. Our results indicate a proportional increase in serum IL-19 concentration among patients with varying degrees of severity, further supporting the role of inflammation as a major etiopathological process in AV patients. Consequently, we can conclude that measuring IL-19 concentration serologically may reflect disease severity in AV patients and could be utilized for patient follow-up. Moreover, our study found no significant changes in acne severity with regard to gender. However, we observed a notable negative correlation between disease severity and the age of participants, indicating that younger age groups are more prone to severe disease due to more aggressive inflammation, as shown in Table 1. Furthermore, our study observed a non-significant change in IL-19 serum concentration in relation to the age and gender of the participants, as shown in Table 2. A similar finding was reported by Mochtar M et al., who also noted a similar relationship between acne severity and patient age [14]. This could be explained by the direct relation of IL-19 levels to the inflammatory process in AV patients, thereby excluding the impact of demographic criteria such as age and gender on IL-19 concentration levels. Given that AV is an inflammatory condition and there is a proportional change in IL-19 concentration with the severity of the condition, coupled with the observed decrease in IL-19 levels following treatment in other inflammatory skin conditions [24, 28], there arises the possibility of treating AV patients by antagonizing the action of IL-19 through various means. The limitations of our study include the small number of participants, the lack of estimation of IL-19 in different types of acne, and the absence of patient follow-up after disease improvement to assess IL-19 levels in response to therapy. Additionally, our study did not estimate the differences in IL-19 concentration following the targeting of IL-19 in vivo with biological therapy.

## Conclusions

In this study, we discovered that IL-19 levels in AV were significantly higher than in matched controls. Among AV patients, serum IL-19 concentration increased proportionally with the severity of acne, suggesting that IL-19 might serve as an inflammatory marker and could potentially relate to AV. Patients with acne scars exhibited significantly higher concentrations of IL-19 in their sera compared to those without scars. Additionally, patients with a history of systemic and topical treatment had higher IL-19 concentrations than those who had not received such treatments. These findings suggest that IL-19 could be used as a laboratory indicator to assess the severity of AV and its tendency to form scars, aiding in disease prognosis.
